# Perceptions and Intention to Get Vaccinated against Mpox among the LGBTIQ+ Community during the 2022 Outbreak: A Cross-Sectional Study in Peru

**DOI:** 10.3390/vaccines11051008

**Published:** 2023-05-21

**Authors:** Julieta M. Araoz-Salinas, Brando Ortiz-Saavedra, Linda Ponce-Rosas, David R. Soriano-Moreno, Anderson N. Soriano-Moreno, Jorge Alave, Jose A. Gonzales-Zamora

**Affiliations:** 1 Peruvian American Medical Society, Albuquerque, NM 87111, USA; julieta.araoz@unmsm.edu.pe (J.M.A.-S.); jxg1416@med.miami.edu (J.A.G.-Z.); 2 Universidad Nacional de San Agustín de Arequipa, Arequipa 04001, Peru; jortizs@unsa.edu.pe; 3 Department of Medicine, Hamilton Medical Center, Dalton, GA 30720, USA; entrega.resultados@markham.edu.pe; 4 Unidad de Investigación Clínica y Epidemiológica, Escuela de Medicina, Universidad Peruana Unión, Lima 15464, Peru; davidsoriano@upeu.edu.pe (D.R.S.-M.); andersonsoriano@upeu.edu.pe (A.N.S.-M.); 5 Departamento de Medicina, Facultad de Medicina, Universidad Peruana Unión, Lima 15464, Peru; 6 Clinica Good Hope, Lima 15074, Peru; 7 Division of Infectious Diseases, Department of Medicine, Miller School of Medicine, University of Miami, Miami, FL 33136, USA

**Keywords:** monkeypox, vaccination, public health, perception, intention, bisexuality, homosexuality, Peru

## Abstract

Vaccination against mpox can control the outbreak by targeting high-risk groups such as the LGBTIQ+ community. The aim of the study was to evaluate the perceptions and intentions to get vaccinated against mpox among the LGBTIQ+ community in Peru. We conducted a cross-sectional study from 1 November 2022 to 17 January 2023 in Peru. We included individuals over 18 years old, belonging to the LGBTIQ+ community, and residing in the departments of Lima and Callao. To evaluate the factors associated with the intention to be vaccinated, we used Poisson regression with robust variance to create a multivariate model. The study comprised 373 individuals who self-identified as members of the LGBTIQ+ community. The participants had a mean age of 31 years (SD ± 9), with 85.0% males and 75.3% reporting to be homosexual men. The majority (88.5%) expressed their intention to receive the vaccine against mpox. Believing that the vaccine is safe was associated with a higher intention to be vaccinated (aPR: 1.24; 95% CI: 1.02 to 1.50; *p* = 0.028). Our study population showed a high level of mpox vaccination intent. Educational campaigns reinforcing the concept of vaccine safety should be conducted to increase the intention and possibly the vaccination rate in the LGBTIQ+ community.

## 1. Introduction

Mpox, previously called monkeypox, is a reemerging zoonotic disease [1], endemic in the Congo basin countries of Africa, that was initially reported in humans in the 1970s. The first reported cases outside Africa occurred in 2003 in the United States [2]. As of 2018, cases were described in Israel, the UK, Singapore, and the USA [3]. In May 2022, there was a precipitous increase in the number of mpox cases reported in Europe and the Americas [3], for which it was declared a global health emergency by the World Health Organization (WHO) on 23 July 2022 [4]. In the current outbreak, according to Centers for Disease Control and Prevention (CDC) data through 29 March 2023, 86,746 cases and 112 deaths from mpox have been reported in 110 countries [5].

Vaccination against human smallpox has been considered a control measure against mpox because both viruses have antigenic similarity, and cross-immunization can be generated [6]. Currently, the WHO proposes the use of three different vaccines against mpox (MVA-BN, ACAM2000, and LC16) [7]; however, MVA-BN is the only vaccine approved by the Food and Drug Administration (FDA) for the prevention of mpox in adults older than 18 years at high risk of mpox infection [8]. In the current outbreak, the most affected group has been men who have sex with men (MSM), a population also at high risk of other sexually transmitted infections, such as HIV [9]. Among them, the risk of HIV infection is 26 times when compared to the general population [10]. Previous studies have reported that individuals with uncontrolled HIV had worse outcomes in the course of mpox [11,12,13]; in this regard, the case series by Mitjà et al. [13] showed that severe complications in mpox were more frequent in HIV patients with a CD4 cell count of <100/mm^3^, and all deaths occurred in HIV patients with a CD4 cell count of <200/mm^3^. Although the CDC suggests the use of tecovirimat for the treatment of mpox under the Expanded Access to Investigational New Drugs (EA-IND) protocol [14], there is currently no quality evidence on the efficacy and safety of the various drug treatments against mpox [15,16]. It has even been hypothesized that resistance to tecovirimat could develop in patients at risk of receiving a prolonged regimen, such as patients coinfected with HIV and mpox. [17]. Therefore, the development and implementation of prevention strategies against mpox in the MSM population are of high importance to avoid significant sequelae and fatal outcomes.

Furthermore, vaccination against mpox in the principal risk group such as gay, bisexual, and MSM has been one of the most important strategies to control the current mpox outbreak [18]. From 31 July to 1 October 2022, among vaccine-eligible men aged 18–49 years in 43 US jurisdictions, mpox incidence among unvaccinated individuals was estimated to be 10 times higher than that reported for fully vaccinated people and 7 times higher than the incidence described for those who received only the first vaccine dose [19]. Few studies have investigated the perception and acceptance of the vaccination against mpox in this high-risk group around the world. There is one study in the Netherlands in 2022 that revealed that 81.5% of gays, bisexuals, and MSM agreed with receiving the mpox vaccine [20], while another study conducted in China in the MSM population reported that 90.2% also agreed [21]. However, there are no studies in Latin America focusing on the acceptance of mpox vaccination in the MSM population, despite the high incidence reported in the Americas, making 87.8% of cases worldwide by February 2023. Globally, among the top 10 countries with the most cases of mpox infection, Peru (n = 3752) was in seventh position after Brazil, Colombia, and Mexico [22].

In Peru, the first case of mpox was described on 26 June 2022 [23]. Nationwide, 3785 confirmed cases and 20 deaths have been reported as of 19 March 2023 [24]. The vaccination process against this disease started in November 2022 in Peru, prioritizing people living with HIV in the first phase [25]. In the second phase, citizens at high risk of contracting sexually transmitted infections, such as MSM, transgender women, and sex workers, were included; in this phase, healthcare workers were also eligible for vaccination [25]. According to WHO, the 3’C model (confidence, complacency, and convenience) represents several factors that work together to influence vaccination decisions [26]. To determine which factors are important to improve, it is necessary to conduct studies on mpox vaccine uptake and whether these might have led to a change in vaccination intention in the population at high risk for mpox. Therefore, it is crucial to focus on evaluating the perceptions and mpox vaccination acceptance in the group at the highest risk such as the LGBTIQ+ community (L, lesbian; G, gay; B, bisexual; T, trans, I, intersexual; Q, queer; +, others) with the purpose of designing public health strategies to resolve doubts and fears about the vaccine. In this cross-sectional study, we evaluated the perception and intention to be vaccinated against mpox among the LGBTIQ+ community during the 2022 outbreak in Peru. We also determined the factors that are independently associated with vaccination intent.

## 2. Materials and Methods

### 2.1. Study Design, Setting, and Participants

We conducted a cross-sectional study from 1 November 2022 to 17 January 2023 in Peru. We adhered to the STROBE checklist for cross-sectional studies (Supplementary Material S1). At the start of data collection, there were 1898 reported cases of mpox in the country, which increased to 3728 by the end of the study period [24]. In response to the outbreak, the Peruvian Ministry of Health initiated free mpox vaccinations on 7 November 2022 [25]. Eligible participants for our study included individuals over 18 years old, self-identified as members of the LGBTIQ+ community, residing in the departments of Lima or Callao, not previously vaccinated against mpox, and not enrolled in an mpox clinical trial.

### 2.2. Sample and Diffusion

Due to the exploratory nature of the study, we determined the sample size based on a 50% intention to be vaccinated against mpox, a 95% confidence level, and a 5% margin of error. Using these parameters, the targeted sample size was 385 individuals. In addition, we considered 5% of possible surveys that did not meet the inclusion criteria or were incorrectly completed, so the minimum target number of completed surveys was 404. We used nonprobabilistic snowball sampling. We distributed the survey through various social media platforms, including Facebook, Twitter, Instagram, and WhatsApp. In order to recruit more participants, we used Grinder, which is a social networking and online dating application targeted toward members of the gay, bisexual, transgender, and queer community. In addition, we administered the survey in person to individuals who attended the clinics of three community-based organizations: “Voluntades Lima Norte”, “Diversex Lima Este”, and “Plan Camino Lima Centro” located in the department of Lima. These organizations focus on preventing sexually transmitted infections in the LGBTIQ+ community.

### 2.3. Questionnaire

The study utilized both online and printed surveys to gather data from participants (Supplementary Material S2). The online survey was created using Google Forms. Meanwhile, participants surveyed in person received a printed survey. The survey sections were as follows: (1) Informed consent, (2) Inclusion criteria, (3) Sociodemographic data, (4) Perceptions about the risk of mpox infection, and (5) Perceptions about the mpox vaccine. To measure perceptions, we employed similar surveys utilized in other countries to assess the intention of the LGBTIQ+ population to get vaccinated against mpox [27,28]. Additionally, we adapted the questions to the Peruvian reality and incorporated specific concepts of the current outbreak in the country. This survey was validated by experts in the field of infectious diseases from Peru and the United States of America.

### 2.4. Independent Variables

We considered the following independent variables: age, area of residence, healthcare worker status, education level, gender, sexual orientation, number of sexual partners in the last 3 months, HIV infection, sexually transmitted infection (other than HIV) in the last 3 months, knowledge of the current outbreak, history of mpox, perceptions toward the risk of infection with mpox, and perceptions regarding the mpox vaccine.

### 2.5. Dependent Variable

To define the variable “intention to be vaccinated against mpox”, we used the following question: “Do you plan to get vaccinated against mpox when the vaccine becomes available?” This question had five response options: 1. “I will get vaccinated,” 2. “It is likely that I will get vaccinated,” 3. “It is very unlikely that I will get vaccinated,” 4. “It is likely that I will not get vaccinated,” and 5. “I will not get vaccinated.” If the respondent answered option 1 or 2, they were considered to have the intention to get vaccinated. If they answered options 3, 4, or 5, they were considered to have no intention to get vaccinated.

### 2.6. Statistical Analysis

The statistical analysis was conducted using R software version 4.2.2. We described participant characteristics using frequencies for categorical variables and mean with standard deviation for numeric variables. In the bivariate analysis, we compared characteristics between the participants who had the intention to be vaccinated and those who did not. We used the Wilcoxon rank-sum test, Fisher’s exact test, and chi-square test of independence depending on the nature of the variable. Finally, we used Poisson regression with robust variance to create a multivariate model that included all variables with a *p*-value < 0.20 in the bivariate analysis. Variables with a *p*-value < 0.05 were considered statistically significant associated factors.

### 2.7. Ethical Aspects

The present study was approved by the institutional ethics committee of the Universidad Peruana Unión (Approval 2022-CE-FCS-UPeU-157) and was registered in the Proyectos de Investigación en Salud (PRISA, by its Spanish acronym) database of the Peruvian National Institute of Health. At the beginning of the survey, informed consent was requested from each participant, the survey was anonymous, and the data obtained were confidential.

## 3. Results

**Figure 1:**
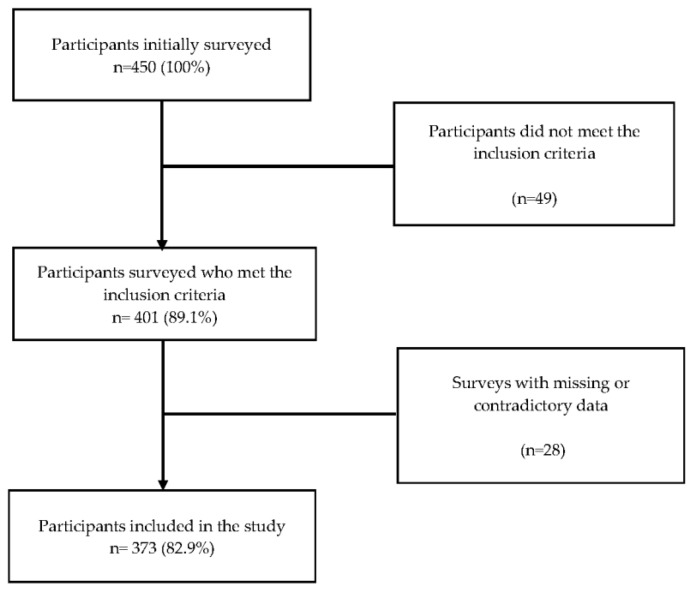
Participant selection flowchart.

A survey was conducted among 450 participants (272 virtual surveys and 178 in-person surveys), of whom 49 individuals were excluded due to not meeting the inclusion criteria, and 28 participants had incomplete or conflicting survey data. Finally, our analysis was carried out with data collected from 373 (237 virtual surveys and 136 in-person surveys) participants who identified themselves as members of the LGBTIQ+ community (Figure 1).

The study sample comprised participants with an average age of 31 (SD: 9) years; most of them resided in central Lima (29.2%) and held university or higher education degrees (61.1%). In terms of gender, the majority self-identified as male (85.0%), while smaller proportions self-identified as female (6.2%), queer (3.2%), transexual (1.3%), transgender female (3.8%), and transgender male (0.5%). Regarding sexual orientation, the majority self-identified as homosexual men (75.3%), while smaller proportions self-identified as bisexual (14.7%), lesbian (3.2%), heterosexual (1.9%), pansexual (3.8%), and other (1.1%). In addition, 35.1% reported being infected with HIV, and 24.9% had a sexually transmitted infection other than HIV within the past 3 months (Table 1).

**Figure 2:**
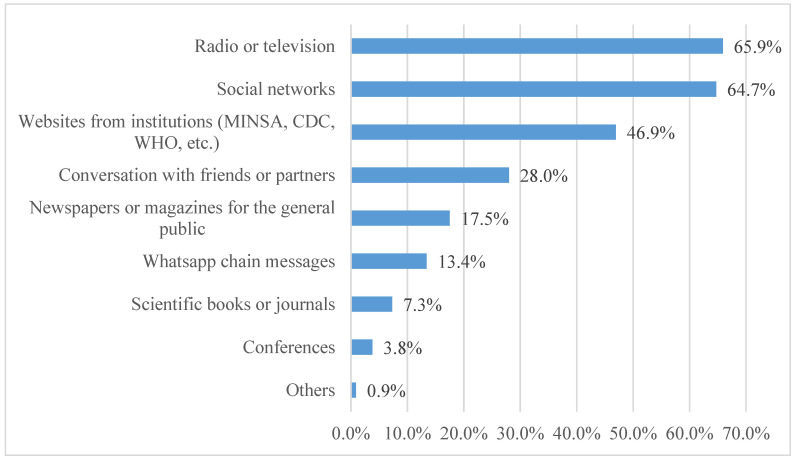
Sources of information on mpox in the Peruvian LGBTIQ+ population (n = 343).

The majority (92.0%) were aware of the mpox outbreak, with 11.8% reporting having experienced the disease. In terms of sources of information about mpox, the most common sources were television or radio (65.9%), social networks (64.7%), websites from official health institutions (MINSA, CDC, WHO, etc.) (46.9%), and conversations with friends (28.0%) (Figure 2).

**Figure 3:**
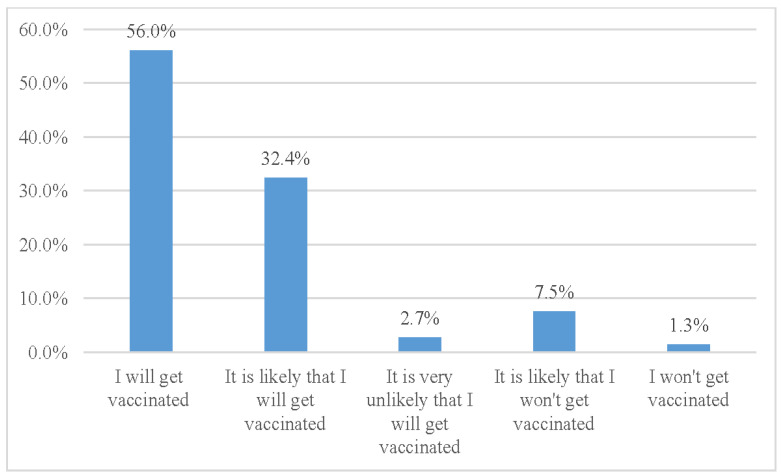
Intention of the LGBTIQ+ population to be vaccinated against mpox.

In terms of intention to be vaccinated against mpox, we found that 56.0% reported that they would get vaccinated, while an additional 32.4% reported that they would likely get vaccinated. A minority of participants reported that they would likely not get vaccinated or would not receive the vaccine (Figure 3).

Furthermore, 54.4% of the participants reported that they would get vaccinated immediately. On the other hand, 32.4% indicated that they would wait to determine whether the vaccine was safe before being vaccinated. A smaller proportion (7.2%) expressed they would only consider being vaccinated if it were mandatory, while 0.8% reported that they would never get vaccinated. Additionally, 5.1% of the respondents reported that they had not yet decided on vaccination.

The intention to be vaccinated was significantly higher among participants who lived in South Lima (*p* = 0.029), had a university degree or higher education level (*p* = 0.022), did not report a history of sexually transmitted infection in the last 3 months (*p* = 0.048), believed that mpox was a highly contagious disease (*p* < 0.001), who were afraid of contracting mpox (*p* = 0.002), believed they were at risk of contracting mpox (*p* = 0.011), considered mpox a severe or dangerous disease (*p* < 0.001), thought that having many sexual partners increased the risk of acquiring mpox (*p* = 0.014), believed the vaccine would protect their health (*p* < 0.001), and believed the vaccine would be safe (*p* < 0.001) (Table 1).

In the multivariable model, we observed a positive association between the belief that the vaccine was safe and the intention to get vaccinated against mpox, which was statistically significant (aPR: 1.24; 95% CI: 1.02 to 1.50; *p* = 0.028) (Table 2). The rest of the variables included in the model did not achieve statistical significance.

## 4. Discussion

This is the first study in Latin America that targeted the LGBTIQ+ community to determine perceptions about mpox and vaccination intent. The overall intention to get vaccinated against mpox in the LGBTIQ+ community in our study was 88.4%, which is one of the highest reported in the literature. One of the first studies on the LGBTIQ+ community was conducted by Wang et al. in the Netherlands, who reported a high intention to get vaccinated (70%). This study was conducted before the start of targeted mpox vaccination in the Netherlands and included 394 MSM [29]. These findings were in line with another Dutch study conducted around the early roll-out of pre-exposure mpox vaccination, which showed an 81.5% vaccination acceptance [20]. Furthermore, a large-scale European survey revealed an intention to get vaccinated of 85–90% in northern countries and 83–88% in western countries. This high vaccination acceptance was related to the perception of increased severity and transmission risk for mpox during this outbreak [30]. In addition, studies with a similar design were conducted in the United Kingdom [31] and China [21], which also reported a high rate of vaccination acceptance of 86% and 90.2%, respectively. According to these studies, the LGBTIQ+ community has a high intention to get vaccinated against mpox, which was also observed in our study.

Regarding the factors independently associated with the intention to be vaccinated against mpox in the LGBTIQ+ community, we found that 85.3% (n = 318) of the respondents considered the mpox vaccine to be safe. Furthermore, in adjusted multivariate analysis, believing that the vaccine was safe increased the intention of being vaccinated by 24%. There are few studies evaluating the association between the perception of safety and vaccination intent. In a study conducted in the Netherlands, it was reported that 45.1% of people eligible for pre-exposure vaccination (PPV) and 45% of people for nonpre-exposure vaccination (non-PPV) believed that the vaccine has no unpleasant adverse effects, and this factor increased the willingness to be vaccinated against mpox [20]. In a study conducted in Ghana on the general population, one of the main determinants of vaccine acceptance was confidence in the vaccine, which increased the odds of mpox vaccine acceptance (aOR: 2.45, 95% CI, 1.93–3.15, *p* < 0.001) [32]. Furthermore, in a study from China, one of the predictors of willingness to get the mpox vaccine among MSM living with HIV was to believe that the mpox vaccine was safe (aOR: 6.6, CI 95%: 2.7–16.4) [33]. It is worth mentioning that mpox vaccine safety has been demonstrated in prelicensure studies, such as a phase 3 study conducted in 2019 on the modified vaccinia Ankara vaccine [34]. Moreover, the CDC considers MVA-BN a safe vaccine, as it only generates mild adverse effects, such as pain, redness, and itching at the inoculation site. Severe allergic reactions are extremely rare [35]. Several authors have theorized that the high acceptance of the mpox vaccine may be due to the fact the population witnessed a worldwide decline in mortality during the COVID-19 pandemic following the implementation of COVID-19 vaccines [36,37]. We postulate that the good safety profile of COVID vaccines observed in many countries, including Peru, could have contributed to the perception of safety for the mpox vaccine in our study.

Regarding the place of residence, our study showed that the area with the highest intention of vaccination was South Lima. The reason behind that may have been that the vaccination campaign was initiated in East and South Lima, so people in those areas were probably better informed about the safety profile and efficacy of the vaccine [25,38]. Other studies have also reported differences in vaccination intent depending on the place of residence. Ahmed et al. [39] revealed that there were significant differences in knowledge and acceptance in the Kurdistan region of Iraq. Kurdistan is divided into four regions: Sulaymaniyah, Erbil, Duhok, and Halabja, where Sulaymaniyah demonstrated the highest level of knowledge, positive attitude, and concern toward mpox.

In the bivariate analysis of our study, we observed that mpox vaccination intent was significantly higher among participants who believed that mpox was a highly contagious disease (*p* < 0.001). This important finding has not been reported in the current literature and should be considered in future studies on mpox vaccine acceptance. Mpox is a contagious disease that is transmitted primarily through sexual contact [40]; however, transmission can also occur through other exposures, including nonsexual contact with active skin lesions and less commonly via direct contact (face to face) with saliva or upper respiratory secretions [41,42]. Of note, mpox is less contagious than smallpox and usually causes a less severe illness [43].

We also found a higher vaccination intent in participants who believed they were at higher risk of contracting mpox. A recent study performed in France showed that among MSM on pre-exposure prophylaxis (PrEP) or living with HIV, 54% of participants felt at risk of being infected with mpox. The authors also found that feeling at risk was an independent determinant of vaccine acceptance [44]. It is important to know that the current data have demonstrated that gay, bisexual, and other men who have sex with men (MSM) made up the majority of cases in the current mpox outbreak [18]. A case series across 16 countries identified several distinct features of the early 2022 mpox outbreak, in which 95% of the transmission was sexual contact and mostly in gay or bisexual men [45]. In Peru, 71.5% of mpox cases were mainly MSM and 55% living with HIV [24]. In addition, of all the patients hospitalized with mpox, 94% were MSM [46].

The present study also revealed that the intention to be vaccinated against mpox was higher in people who believed that the vaccine would protect their health (*p* < 0.001). These findings are similar to those reported by Ghazy et al. [32], who found that confidence was significantly associated with mpox vaccine acceptance in the Ghanaian population. In their study, they defined confidence as “trust in the vaccine’s dependability and effectiveness, in the health system, and the healthcare personnel” [32]. The vaccines proposed by WHO for mpox were initially designed for the prevention of smallpox [7], so evidence of their efficacy and effectiveness against mpox at the beginning of the current outbreak was sparse. However, observational studies on the effectiveness of the MVA-BN vaccine have been recently published. Payne et al. [47] analyzed weekly reports from 43 US jurisdictions and found that the incidence of mpox in unvaccinated adult men was 7,4-fold higher compared with adult men who received only one dose of MVA-BN vaccine and 9,6-fold higher compared with men who received two doses. Similarly, Wolff Sagy et al. [48] conducted a retrospective analysis in 2054 Israeli adults with risk factors for mpox infection, in which they found that vaccination with one dose of MVA-BN was associated with an 86% reduction in the risk of mpox. These real-world data demonstrate that the MVA-BN vaccine effectively protects against mpox infection.

Another important finding of our study was that mpox vaccination intent was higher among respondents who considered mpox a severe or dangerous disease (*p* < 0.001). Before the current mpox outbreak, WHO reported mpox mortality rates of up to 11% [49]. In addition, at the beginning of the current outbreak, different studies reported that the main affected group was MSM [50], who have a higher HIV prevalence compared to the general population [10] and were the predominant group of reported cases with mpox and HIV coinfection [45,51,52]. In response, in July 2022, the Peruvian government issued a guideline for the prevention and management of patients with mpox, which considered HIV+ and immunosuppressed patients at risk of developing more severe disease and mpox complications [53]. Likewise, the Ministry of Health of Peru organized multiple information campaigns on the prevention of mpox infection, mainly among vulnerable populations, such as people with HIV and MSM [54]. These data provided by the Ministry of Health during the current outbreak alerted the LGBTIQ+ community and may explain our results.

It is important to note that most participants in our study (92.0%) were aware of the mpox outbreak. The main source of information was television or radio (65.9%), followed by social networks (64.7%) and web pages of official health institutions (MINSA, CDC, WHO, etc.) (46.9%). The dissemination of truthful information with simple and easy-to-understand language by competent entities is key, as this factor probably contributed to the high acceptance of the mpox vaccine. However, when asked about the information provided by the Peruvian Ministry of Health, only 30.8% of participants claimed that this institution was adequately informing about mpox, which is concerning because it may lead to the development of conspiracy theories about the vaccine in the community [55]. This is clearly an aspect that the central government should improve—providing transparency and effective factual correction on the fake news spread through different media.

### Limitations and Strengths

Our study has some limitations. Despite having a good sample size, it is not representative of the LGBTIQ+ population from Peru because we used nonprobability snowball sampling. In addition, it is possible that there was a response bias, as respondents probably wanted to give a socially accepted answer [56] in the context of the disinformation campaigns carried out in Peru [57]. Finally, most of the surveys were online, where there was no face-to-face control of the completion, so different factors could have affected the veracity of the answers given, such as the search for information and the completion of the same survey by two or more people. Regarding strengths, to our knowledge, this study is the first one in Latin America to assess mpox vaccination intent among the LGBTIQ+ community during the 2022 mpox outbreak. In addition, the survey (beginning 1 November) was conducted at the beginning of the vaccination campaign in Peru on 7 November 2022 [25], which would probably reflect more accurately a defined opinion of the participants regarding their willingness to receive the mpox vaccine [58].

## 5. Conclusions

Our study showed a high intention to be vaccinated against mpox among the LGBTIQ+ community in the departments of Lima and Callao in Peru, a finding that is related to the perception of this population to be at higher risk of contracting mpox, which is believed to be a severe and very contagious disease according to the study participants. We found that the perception of safety for the mpox vaccine was the only factor independently associated with the intention to be vaccinated; thus, this factor should be considered and reinforced in educational campaigns, which could potentially lead to an increase in the rate of vaccination. Despite the high percentage of mpox vaccine awareness found in our study, there are still concerns about information adequacy provided by the central government, which is a problem that should be addressed to avoid the development of conspiracy theories and misconceptions about the vaccine, especially in areas where the acceptance has been lower, such as Callao and East Lima.

## Figures and Tables

**Table 1:** Comparison of characteristics between respondents with intention to be vaccinated and those who did not intend to be vaccinated (n = 373).

Characteristic	n = 373	No Intention to Be Vaccinated	Intention to Be Vaccinated	*p*-Value *
		n = 43	n = 330	
Age, mean (SD)	31 (9)	29 (8)	31 (9)	0.111
Zone of residence				**0.029**
Callao	28 (7.5%)	5 (17.9%)	23 (82.1%)	
Central Lima	109 (29.2%)	9 (8.3%)	100 (91.7%)	
East Lima	89 (23.9%)	10 (11.2%)	79 (88.8%)	
North Lima	101 (27.1%)	18 (17.8%)	83 (82.2%)	
South Lima	46 (12.3%)	1 (2.2%)	45 (97.8%)	
Healthcare worker				0.838
No	326 (87.4%)	38 (11.7%)	288 (88.3%)	
Yes	47 (12.6%)	5 (10.6%)	42 (89.4%)	
Education level				**0.022**
High school	78 (20.9%)	13 (16.7%)	65 (83.3%)	
Technical	56 (15.0%)	9 (16.1%)	47 (83.9%)	
College/university	228 (61.1%)	18 (7.9%)	210 (92.1%)	
None of the above	11 (2.9%)	3 (27.3%)	8 (72.7%)	
Gender				0.053
Male	317 (85.0%)	32 (10.1%)	285 (89.9%)	
Female	23 (6.2%)	4 (17.4%)	19 (82.6%)	
Queer	12 (3.2%)	1 (8.3%)	11 (91.7%)	
Other	21 (5.6%)	6 (28.6%)	15 (71.4%)	
Sexual Orientation				0.340
Homosexual men	281 (75.3%)	30 (10.7%)	251 (89.3%)	
Bisexual	55 (14.7%)	6 (10.9%)	49 (89.1%)	
Other	37 (9.9%)	7 (18.9%)	30 (81.1%)	
Sexual partners in the last 3 months, mean (SD)	6 (10)	7 (8)	6 (10)	0.156
HIV infection				0.475
No	242 (64.9%)	30 (12.4%)	212 (87.6%)	
Yes	131 (35.1%)	13 (9.9%)	118 (90.1%)	
Sexually transmitted infection in the last 3 months (other than HIV)				**0.048**
No	280 (75.1%)	27 (9.6%)	253 (90.4%)	
Yes	93 (24.9%)	16 (17.2%)	77 (82.8%)	
Knowledge about the current mpox outbreak				0.999
No	30 (8.0%)	3 (10.0%)	27 (90.0%)	
Yes	343 (92.0%)	40 (11.7%)	303 (88.3%)	
History of mpox disease				0.141
No	329 (88.2%)	35 (10.6%)	294 (89.4%)	
Yes	44 (11.8%)	8 (18.2%)	36 (81.8%)	
**Perceptions about mpox**				
Do you think mpox is a very contagious disease?				**<0.001**
No	49 (13.1%)	15 (30.6%)	34 (69.4%)	
Yes	324 (86.9%)	28 (8.6%)	296 (91.4%)	
Mpox is transmitted by direct contact with the skin lesions of a sick person.				0.103
Agree	335 (89.8%)	35 (10.4%)	300 (89.6%)	
Neither agree nor disagree	30 (8.0%)	7 (23.3%)	23 (76.7%)	
Disagree	8 (2.1%)	1 (12.5%)	7 (87.5%)	
Mpox can be transmitted by talking to a sick person.				0.875
Agree	123 (33.0%)	13 (10.6%)	110 (89.4%)	
Neither agree nor disagree	69 (18.5%)	9 (13.0%)	60 (87.0%)	
Disagree	181 (48.5%)	21 (11.6%)	160 (88.4%)	
Mpox is transmitted by semen.				0.734
Agree	194 (52.0%)	20 (10.3%)	174 (89.7%)	
Neither agree nor disagree	114 (30.6%)	15 (13.2%)	99 (86.8%)	
Disagree	65 (17.4%)	8 (12.3%)	57 (87.7%)	
Mpox is transmitted by saliva.				0.141
Agree	199 (53.4%)	17 (8.5%)	182 (91.5%)	
Neither agree nor disagree	101 (27.1%)	16 (15.8%)	85 (84.2%)	
Disagree	73 (19.6%)	10 (13.7%)	63 (86.3%)	
Mpox is a disease that mainly affects gay or bisexual men.				0.293
Agree	138 (37.0%)	19 (13.8%)	119 (86.2%)	
Neither agree nor disagree	81 (21.7%)	11 (13.6%)	70 (86.4%)	
Disagree	154 (41.3%)	13 (8.4%)	141 (91.6%)	
Fear of contracting mpox disease.				**0.002**
No	107 (28.7%)	21 (19.6%)	86 (80.4%)	
Yes	266 (71.3%)	22 (8.3%)	244 (91.7%)	
Do you think you are at risk of contracting mpox?				**0.011**
Agree	211 (56.6%)	18 (8.5%)	193 (91.5%)	
Neither agree nor disagree	99 (26.5%)	11 (11.1%)	88 (88.9%)	
Disagree	63 (16.9%)	14 (22.2%)	49 (77.8%)	
Do you believe mpox is a serious or dangerous disease?				**<0.001**
No	125 (33.5%)	24 (19.2%)	101 (80.8%)	
Yes	248 (66.5%)	19 (7.7%)	229 (92.3%)	
Condoms prevent the spread of mpox.				0.164
Agree	116 (31.1%)	17 (14.7%)	99 (85.3%)	
Neither agree nor disagree	94 (25.2%)	13 (13.8%)	81 (86.2%)	
Disagree	163 (43.7%)	13 (8.0%)	150 (92.0%)	
Having many sexual partners increases the risk of contracting mpox.				**0.014**
Agree	304 (81.5%)	28 (9.2%)	276 (90.8%)	
Neither agree nor disagree	42 (11.3%)	9 (21.4%)	33 (78.6%)	
Disagree	27 (7.2%)	6 (22.2%)	21 (77.8%)	
The Ministry of Health of Peru is adequately informing the population about mpox.				0.975
Agree	115 (30.8%)	13 (11.3%)	102 (88.7%)	
Neither agree nor disagree	99 (26.5%)	11 (11.1%)	88 (88.9%)	
Disagree	159 (42.6%)	19 (11.9%)	140 (88.1%)	
**Perceptions about the mpox vaccine**				
Is there a vaccine to prevent mpox?				0.115
No	20 (5.4%)	4 (20.0%)	16 (80.0%)	
I don’t know	108 (29.0%)	16 (14.8%)	92 (85.2%)	
Yes	245 (65.7%)	23 (9.4%)	222 (90.6%)	
Do you think that the vaccine against mpox would protect your health?				**<0.001**
No	51 (13.7%)	18 (35.3%)	33 (64.7%)	
Yes	322 (86.3%)	25 (7.8%)	297 (92.2%)	
How safe do you think the mpox vaccine would be?				**<0.001**
Not safe	55 (14.7%)	19 (34.5%)	36 (65.5%)	
Safe	318 (85.3%)	24 (7.5%)	294 (92.5%)	

SD, standard deviation. * Statistically significant *p*-values are in bold.

**Table 2:** Multivariable analysis of factors associated with the intention to get vaccinated.

Characteristic	Multivariable Model		
	aPR	95% CI	*p*-Value *
Age, mean (SD)	1.00	1.00–1.01	0.108
Zone of residence in Lima			
Callao	Ref.		
Central	1.01	0.86–1.18	0.910
East	1.01	0.86–1.19	0.888
North	0.96	0.81–1.13	0.614
South	1.10	0.94–1.27	0.240
Education level			
High school	Ref.		
Technical	0.97	0.84–1.11	0.647
College/university	1.02	0.93–1.12	0.676
None of the above	1.00	0.71–1.41	0.987
Gender			
Male	Ref.		
Female	0.97	0.82–1.15	0.723
Queer	0.89	0.71–1.11	0.289
Other	1.03	0.88–1.22	0.696
Sexual partners in the last 3 months	1.00	1.00–1.00	0.484
Sexually transmitted infection in the last 3 months (other than HIV)			
No	Ref.		
Yes	0.97	0.89–1.05	0.453
History of mpox disease			
No	Ref.		
Yes	0.94	0.83–1.07	0.363
Do you think mpox is a very contagious disease?			
No	Ref.		
Yes	1.13	0.95–1.34	0.179
Mpox is transmitted by direct contact with the skin lesions of a sick person.			
Agree	Ref.		
Neither agree nor disagree	0.98	0.80–1.19	0.807
Disagree	1.02	0.83–1.26	0.839
Mpox is transmitted by saliva.			
Agree	Ref.		
Neither agree nor disagree	0.98	0.89–1.08	0.726
Disagree	0.96	0.88–1.06	0.430
Fear of contracting mpox disease.			
No	Ref.		
Yes	1.01	0.92–1.12	0.805
Do you think you are at risk of contracting mpox?			
Agree	Ref.		
Neither agree nor disagree	1.04	0.95–1.14	0.423
Disagree	0.89	0.77–1.02	0.090
Do you believe mpox is a serious or dangerous disease?			
No	Ref.		
Yes	1.07	0.98–1.16	0.109
Condoms prevent the spread of mpox.			
Agree	Ref.		
Neither agree nor disagree	0.96	0.86–1.06	0.408
Disagree	0.98	0.90–1.07	0.632
Having many sexual partners increases the risk of contracting mpox.			
Agree	Ref.		
Neither agree nor disagree	0.97	0.81–1.16	0.746
Disagree	1.07	0.89–1.28	0.480
Is there a vaccine to prevent mpox?			
No	Ref.		
I don’t know	0.98	0.79–1.23	0.889
Yes	0.98	0.79–1.21	0.842
Do you think that the vaccine against mpox would protect your health?			
No	Ref.		
Yes	1.21	0.99–1.47	0.063
How safe do you think the mpox vaccine would be?			
Not safe	Ref.		
Safe	1.24	1.02–1.50	0.028

aPR, adjusted prevalence ratio; CI, confidence interval; Ref., reference. * Statistically significant *p*-values are in bold.

## Data Availability

Research data are not available due to privacy.

## Supplementary Materials

The following supporting information can be downloaded at: https://www.mdpi.com/article/10.3390/vaccines11051008/s1. Supplementary material S1-STROBE Statement-Checklist of items that should be included in reports of cross-sectional studies; Supplementary material S2-Survey to evaluate the perceptions and intention to get vaccinated against mpox.
