# Supplementary material for: Perceptions and Intention to Get Vaccinated against Mpox among the LGBTIQ+ Community during the 2022 Outbreak: A Cross-Sectional Study in Peru
Source: Vaccines (Basel). 2023 May 21;11(5):1008. doi: 10.3390/vaccines11051008 (PMC10220940; doi:10.3390/vaccines11051008)
Supplement: Supplementary file 1 [file vaccines-11-01008-s001.zip › Informed consent (for vaccines).pdf]

Informed consent

Dear participant, I am Dr. Jose Gonzales-Zamora Infectious Disease Physician from Miami University and volunteer professor from Universidad Peruana Unión. This survey has the purpose to study the Perception and Intention to Vaccinate against Mpox among LGTBIQ+ Community during the 2022 outbreak. This information will be useful to develop educational programs to resolve doubts about the Monkeypox vaccination.

Your participation is totally free. If you wish to participate, please respond all the questions. You can leave the survey at any moment if you do not want to continue. Be aware that this survey is totally anonymous. We don't ask for any personal information to identify the participant.

This study has the approval of the Ethical Committed from Universidad Peruana Union (Number: 2022-CE-FCS - UPeU-157) and it is registered in PRISA (Proyectos de Investigacion en Salud) from Minister Health of Peru.

To be able for participating, you must have the following requirements:

Living in Lima or Callao
More than 18 years old.
To be member of the LGTBIQ+ Community (L: Lesbian, G: Gay, T: Trans, B: Bisexual, I: Intersex, Q: Queer, +: Others).

If you received the Monkeypox vaccine or you are participating in research related to monkeypox vaccine, you are not eligible to participle in this study.
