# Supplementary material for: Perceptions and Intention to Get Vaccinated against Mpox among the LGBTIQ+ Community during the 2022 Outbreak: A Cross-Sectional Study in Peru
Source: Vaccines (Basel). 2023 May 21;11(5):1008. doi: 10.3390/vaccines11051008 (PMC10220940; doi:10.3390/vaccines11051008)
Supplement: Supplementary file 1 [file vaccines-11-01008-s001.zip › Supplementary material S2 (for vaccines).pdf]

Supplementary material S2. Survey to assess Perception and Intention to Vaccinate against Mpox.

Inclusion Criteria

1. Do you currently live in Lima or Callao?

a. No (survey ends)
b. Yes

2. Do you belong to the LGTBIQ+ Community (L: Lesbian, G: Gay, T: Trans, B: Bisexual, I: Intersex, Q: Queer, +: Others)?

a. No (survey ends)
b. Yes

3. Are you more than 18 years old?

a. No (survey ends)
b. Yes

Exclusion criteria

4. Have you received the Monkeypox vaccine before?

a. No
b. Yes (survey ends)

5. Are you participating in a clinical trial related to the monkeypox vaccine?

a. No
b. Yes (survey ends)

Basic Participant Information

(6) What is your age? (years) …….

(7) Indicate your current place of residence:

Ancón
Ate
Barranco
Breña
Callao-Mi Perú
Callao-Ventanilla
Callao-Callao
Callao-Carmen de la Legua Reynoso
Callao-Bellavista
Callao-La Perla
Callao-La Punta
Carabayllo

Chaclacayo
Chorrillos
Cieneguilla
Comas
El Agustino
Independencia
Jesús María
La Molina
La Victoria
Cercado de Lima
Lince
Los Olivos
Lurigancho
Lurín
Magdalena del Mar
Pueblo Libre
Miraflores
Pachacamac
Pucusana
Puente Piedra
Punta Hermosa
Punta Negra
Rímac
San Bartolo
San Borja
San Isidro
San Juan de Lurigancho
San Juan de Miraflores
San Luis
San Martin de Porres
San Miguel
Santa Anita
Santa María del Mar
Santa Rosa
Santiago de Surco
Surquillo
Villa El Salvador
Villa María del Triunfo

(8) Are you a health professional?

a. No
b. Yes

(9) What is your grade instruction?

a. Primary School
b. Secondary School
c. University
d. Technician
e. None of the above

(10) What is your gender identity?

a. Woman
b. Man
c. Woman transgender (At birth, they were assigned male, but identify as female)
d. Man transgender (At birth, they were assigned female, but identify as male)
e. Transexual (A person who undergoes surgical or hormonal treatment to transition to a different gender identity.)
f. Intersex (A person who does not identify with the notion of male or female.)
g. Queer (A person whose gender does not conform to the established norms of gender expression in society)
h. Other: …………………..

(11) What is your sexual orientation?

a. Lesbian
b. Gay (Homosexual man)
c. Heterosexual
d. Bisexual (A person who feels attraction towards people of the same and different genders/sexes.)
e. Pansexual (A person who can feel attraction towards another person regardless of their sex or gender.)
f. Other: ……………………

(12) In the past three months, how many people have you had sex?

Number: .………………………

(13) Are you an HIV patient?

a. No (Please, go to question number 15)
b. Yes (Please, answer the following question)

(14) Do you receive HIV treatment?

a. No
b. Yes

(15) In the past three months, have you had a sexually transmitted disease? (Such as syphilis, gonorrhea, chlamydia, herpes, etc.)

a. No
b. Yes

(16) Have you heard about the current outbreak of monkeypox?

a. No
b. Yes

(17) What sources have you used to obtain information about monkeypox (you can choose more than one alternative)?

a. Books or scientific journals
b. Conferences
c. Official health institution's websites: CDC, WHO, etc
d. Radio or Television
e. Newspapers or magazines for the general public
f. Social Media (Facebook, Instagram, Twitter, TikTok)
g. WhatsApp chains
h. I have not received any information.

(18) Have you been sick with monkeypox?

a. No
b. Yes

Perception of the risk of Monkeypox Infection.

(19) Do you think that Monkeypox is a very contagious disease?

a. Definitely yes
b. Probably yes
c. Unlikely
d. Very unlikely
e. Definitively no

(20) What do you think about the following statement? Monkeypox is transmitted through direct contact with the skin lesions of a sick person.

a. I totally agree
b. I agree
c. Neither agree nor disagree
d. I disagree
e. I totally disagree

(21) Do you agree with the following statement? Monkeypox can be transmitted by talking with a sick person.:

a. I totally agree
b. I agree
c. Neither agree nor disagree
d. I disagree
e. I totally disagree

(22) Do you agree with the following statement? Monkeypox is transmitted by semen.

a. I totally agree
b. I agree
c. Neither agree nor disagree
d. I disagree

e. I totally disagree

(23) Do you agree with the following statement? Monkeypox is transmitted by saliva.

a. I totally agree
b. I agree
c. Neither agree nor disagree
d. I disagree
e. I totally disagree

(24) What is your opinion on the following statement? Monkeypox is a disease that mainly affects gay or bisexual men.

a. I totally agree
b. I agree
c. Neither agree nor disagree
d. I disagree
e. I totally disagree

(25) Are you afraid of catching Monkeypox disease?

a. No
b. Yes

(26) What is your opinion on the following statement? You are at risk of getting an infection of the monkeypox virus.

a. I totally agree
b. I agree
c. Neither agree nor disagree
d. I disagree
e. I totally disagree

(27) Do you believe that monkeypox is a severe or dangerous disease?

a. Definitely yes
b. Probably yes
c. Unlikely
d. Very unlikely
e. Definitively no

Perception of preventative measures in front of the Monkeypox outbreak

(28) Do you agree with the following statement? Condoms prevent the transmission of monkeypox.

a. I totally agree
b. I agree
c. Neither agree nor disagree
d. I disagree
e. I totally disagree

(29) What is your opinion about the following statement? Having multiple sexual partners increases the risk of getting monkeypox disease.

a. I totally agree
b. I agree
c. Neither agree nor disagree
d. I disagree
e. I totally disagree

(30) Do you agree with the following statement? The Ministry of Health of Peru is adequately informing the population about monkeypox.

a. I totally agree
b. I agree
c. Neither agree nor disagree
d. I disagree
e. I totally disagree

(31) There is a vaccine that can prevent monkeypox.

a. NO
b. YES
c. I don’t know

(32) Do you think that the vaccine against monkeypox would PROTECT your health?
a. Definitely YES, it would protect me.
b. Probably it would protect me.
c. It is unlikely to protect me.
d. Very unlikely to protect me.
e. Definitely NO, it would not protect me.

(33) How SAFE do you believe the vaccine against monkeypox would be?
a. It would be very safe
b. It would be moderately safe
c. It would be slightly safe
d. It would be hardly safe
e. It would be unsafe

(34) Do you plan to vaccinate against Monkeypox when the vaccine is available?
a. I will get vaccinated
b. It is likely that I will get vaccinated
c. It is very unlikely that I will get vaccinated
d. It is likely that I will not get vaccinated
e. I will not get vaccinated

(35) How soon would you get vaccinated against monkeypox when the vaccine is available?
a. I would get vaccinated immediately
b. I would wait some time to know if the vaccine is safe
c. I would only get vaccinated if it were mandatory
d. I haven't decided yet

e. I would never get vaccinated.

- Thank you very much for your participation.
